# Engineering Hepadnaviruses as Reporter-Expressing Vectors: Recent Progress and Future Perspectives

**DOI:** 10.3390/v8050125

**Published:** 2016-05-10

**Authors:** Weiya Bai, Xiaoxian Cui, Youhua Xie, Jing Liu

**Affiliations:** Key Laboratory of Medical Molecular Virology (MOH & MOE) and Institutes of Biomedical Sciences, School of Basic Medical Sciences, Shanghai Medical College, Fudan University, Shanghai 200032, China; bwy.1989@163.com (W.B.); cuixiaoxian9@hotmail.com (X.C.)

**Keywords:** recombinant virus, viral vector, gene delivery, HBV, DHBV

## Abstract

The *Hepadnaviridae* family of small, enveloped DNA viruses are characterized by a strict host range and hepatocyte tropism. The prototype hepatitis B virus (HBV) is a major human pathogen and constitutes a public health problem, especially in high-incidence areas. Reporter-expressing recombinant viruses are powerful tools in both studies of basic virology and development of antiviral therapeutics. In addition, the highly restricted tropism of HBV for human hepatocytes makes it an ideal tool for hepatocyte-targeting *in vivo* applications such as liver-specific gene delivery. However, compact genome organization and complex replication mechanisms of hepadnaviruses have made it difficult to engineer replication-competent recombinant viruses that express biologically-relevant cargo genes. This review analyzes difficulties associated with recombinant hepadnavirus vector development, summarizes and compares the progress made in this field both historically and recently, and discusses future perspectives regarding both vector design and application.

## 1. Introduction

*Hepadnaviridae* is a family of small, enveloped DNA viruses with notable hepatic tropism, especially in mammals, and transmission is achieved predominantly through parenteral routes [1,2]. The viral genome consists of partially double-stranded, relaxed circular DNA (rcDNA) that is produced through a process involving a reverse transcription step similar to retroviruses [2,3]. These features led to the classification of hepadnaviruses under group VII (dsDNA(RT) or pararetrovirus) in the Baltimore system, along with certain similar DNA viruses infecting plants.

Hepadnaviruses usually have highly-restricted host ranges and have traditionally been classified into two genera based on host specificity [4,5]. Orthohepadnaviruses infect mammals, with members including the prototype hepatitis B virus (HBV) of humans, woolly monkey hepatitis B virus (WMHBV), woodchuck hepatitis virus (WHV), and ground squirrel hepatitis virus (GSHV), *etc*. Avihepadnaviruses infect various domesticated and wild birds, with members including the prototype duck hepatitis B virus (DHBV), as well as heron hepatitis B virus (HHBV), *etc*. In recent years, the advent and advances of next-generation sequencing and other metagenomics technologies have enabled the discovery of new HBV-like viruses that infect hosts previously not known to be affected by hepadnaviruses, such as bats [6] and fish [7]. In addition, analyses of whole genome sequencing data have also led to the discovery of endogenous hepadnaviral sequences in genomes of avian [8,9,10] and reptilian [11] species, suggesting a family history spanning millions of years. In light of these recent discoveries, hepadnaviruses, including extant and now extinct ones, are obviously far more diverse than previously understood and the taxonomy may well be expanded and modified in the future.

Among extant hepadnaviruses, orthohepadnaviruses productively infect only hepatocytes of the liver, whereas DHBV has been shown to additionally infect certain other cell types of the liver and non-liver organs [3]. Hepato-tropism has been considered the result of tissue-specific distribution of both receptor(s) required for viral entry and transcription factors required for viral expression [12,13]. Accordingly, liver pathologies including hepatitis are major manifestations of symptomatic hepadnaviral infections in both human and animals [1,3]. However, as hepadnavirus infection is neither cytopathic nor cytolytic, hepatitis is generally considered a consequence of the activated host immune response against infected hepatocytes.

HBV is a major human pathogen and constitutes a severe public health problem in high-incidence areas [5,14]. HBV infection of adults is usually asymptomatic or manifests as self-resolving acute hepatitis, while a small percentage of patients fail to clear the virus and become infected for life. Vertical transmission of HBV from infected mothers to neonates typically results in asymptomatic chronic infection accompanied by immunotolerance towards HBV, which would be broken in later life leading to active hepatitis. Chronic HBV infection is associated with higher risks of cirrhosis and hepatocellular carcinoma (HCC) [1]. Although extensive adoption of preventive HBV vaccine has drastically reduced incidence of new infections, the World Health Organization estimated that HBV chronically infects ~240 million people worldwide and causes about 600,000 related deaths annually [14].

Duck/DHBV and woodchuck/WHV have been used as model systems of HBV infections for decades, and have helped significantly in understanding hepadnavirus virology and developing anti-HBV therapeutics [15]. However, chronic DHBV infection is not associated with liver cirrhosis or HCC in ducks, while WHV-related HCC is mechanistically much more homogenous than HBV-associated human HCC [3], underlining the fact that HBV and human or humanized animal systems are required for studying various important aspects of HBV pathogenesis.

Reverse genetic systems were established for HBV as well as DHBV decades ago and have become standard tools in studies of viral functions as well as virus-host interactions. In contrast, owing to certain characteristics in genome organization and life cycle (see next section), development of reporter-expressing virus systems based on hepadnaviruses has met with far less successes than many other virus families. Nevertheless, recombinant hepadnavirus vector systems could serve as powerful tools for both studying fundamental questions in hepadnavirus virology and evaluating clinical interventions for chronic HBV infection, as have been demonstrated by other recombinant virus systems. In addition, the highly restricted host range and hepato-tropism of HBV makes it a uniquely ideal tool for hepatocyte-targeting applications, such as liver-specific therapeutic gene delivery. This review attempts to summarize difficulties associated with recombinant hepadnavirus vector development and progress made historically and recently, and discuss future perspectives in this field regarding both vector design and application.

## 2. Genome Organization and Life Cycle of Hepadnaviruses

This section will briefly describe aspects of hepadnavirus virology that most pertains to recombinant virus development. For a more detailed discussion of current understandings in this field, readers are referred to comprehensive reviews published recently [3,12,16].

**Figure 1:**
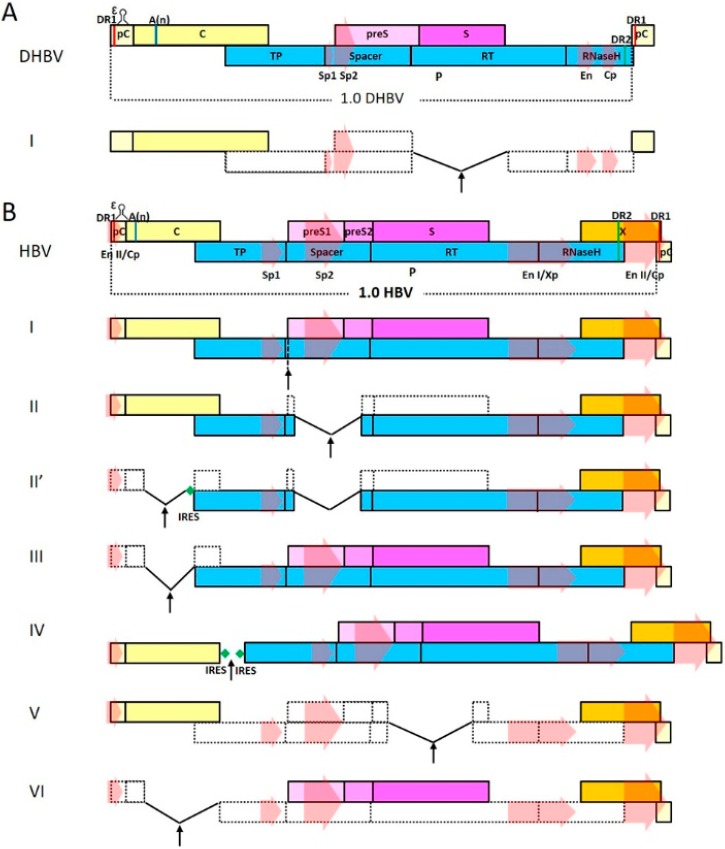
Schematic representation of genome organization of HBV and DHBV and major recombinant (D)HBV vector designs. Terminally-redundant wild-type genomes are shown to reflect the circularity and 1.0 copy of each genome is marked out beginning at the start codon of preC(pC)/C ORF. ORFs are represented by boxes and ORFs destroyed in recombinant vectors are depicted using dotted lines. Promoters (Cp, Sp1, Sp2, and Xp) and enhancers (En, EnI, EnII) are represented by arrows pointing in the direction of transcription. *Cis* elements required for replication and encapsidation are also depicted. ε, encapsidation signal. A(n), polyadenylation signal. DR, direct repeats. (**A**) Wild-type DHBV genome and recombinant DHBV design: I, cargo gene replaces S ORF in-frame and destroys the overlapping P ORF. (**B**) wild-type HBV genome and recombinant HBV designs: I, cargo gene is inserted in-frame with P ORF within spacer region, upstream of preS1 ORF. P ORF may or may not be terminated depending on cargo. II, cargo gene is inserted in a deletion in polymerase spacer in-frame with preS1/preS2 ORF, without terminating the overlapping P ORF. II’, derivative of II. The cargo gene replaces the central part of prematurely-terminated C ORF, followed by IRES upstream of P ORF with a maximized deletion in the spacer region that retains polymerase activity but destroys preS1/preS2 ORF. III, the cargo gene replaces central part of C ORF and may or may not be expressly fused to the remaining N-terminal of preC/C, depending on the design. IV, IRES units are introduced to separate de-overlapped C and P ORFs. No viral ORF is obliterated. V, the cargo gene replaces S ORF in-frame and destroys the overlapping P ORF. VI, the cargo gene replaces the central and C-terminal of C ORF, destroying the overlapping P ORF. Arrows indicate insertion sites. Vector designs with obliterated C and/or P ORFs require *trans*-complementation of the obliterated proteins for genome replication. Vector designs with obliterated envelope ORFs require *trans*-complementation of envelope proteins for production of progeny virus.

All extant hepadnaviruses have a genome length of ~3.0–3.4 kb and nearly identical, highly-compact genome organization (Figure 1). The orthohepadnavirus genome contains four overlapping ORFs that encompass the entire genome: preC/C ORF encodes the nucleocapsid protein C (core, HBcAg) and an N-terminally extended secreted form called e antigen (HBeAg) using two distinct start codons; P ORF encodes the viral polymerase; preS1/preS2/S ORF encodes three co-terminal forms of viral envelope protein named small (S), middle (M), and large (L) surface antigen (HBsAg) that are translated from three distinct start codons; X ORF encodes the X protein (HBx) that plays multiple functions in the viral life cycle and virus-host interactions. Compared to orthohepadnaviruses, avihepadnaviruses harbor a preS/S ORF, instead of preS1/preS2/S, that only encodes two forms of co-terminal envelope proteins, and avihepadnaviral P and preC/C ORFs overlap each other, lacking a conventional X ORF in between (Figure 1A). In addition to overlapping ORFs, hepadnaviral genomes also contain multiple *cis*-acting elements essential for various steps of the viral life cycle: promoters (Cp, Sp1, Sp2, and Xp) and enhancers (EnI and EnII) for transcription, epsilon (encapsidation) signal for initiation of reverse transcription and capsid packaging, direct repeats 1 and 2 (DR1 and DR2) for polymerase translocation during genome replication, *etc*.

Mature, infectious hepadnavirus virions contain the rcDNA genome and enter hepatocytes through interactions between large surface antigens and specific cellular receptor(s). Once inside, the viral capsid releases its genome contents into nucleus, where rcDNA is converted into covalently-closed circular DNA (cccDNA) that then serves as a transcription template for all viral RNA species. Both core and viral polymerase are translated from the same transcript, termed pregenomic RNA (pgRNA), which also serves as a template for genome replication. Most notably, for wild-type viruses, nascent polymerase co-translationally binds to the epsilon signal at the 5′ terminal of its own pgRNA *in cis*, and initiates reverse transcription using an internal, conserved tyrosine residue as a primer for negative-strand DNA synthesis. A vital translocation step then follows, with the polymerase-primer “jumping” from the 5′ terminal to the 3′ terminal of pgRNA, which involves the DR1/DR2 direct repeat sequences at both termini. Packaging of the pgRNA/polymerase complex by viral core proteins is essential for productive genome replication and eventual formation of progeny rcDNA. Capsids containing newly-formed rcDNA then obtain host-derived membranes containing viral envelope proteins to produce progeny viruses and exit through budding at the ER. Alternatively, capsids are rerouted to the nucleus and the released rcDNA are converted into cccDNA to increase the level of transcription templates.

## 3. Hepadnavirus-Specific Difficulties for the Design and Development of Viral Vectors

Characteristic peculiarities in the genome organization and life cycle of hepadnaviruses pose specific difficulties and problems for efforts aimed at engineering recombinant hepadnavirus vectors.

First of all, genome sizes of wild-type viruses very likely represent the maximum limit that could be tolerated by the viral replication and packaging mechanisms. This has been demonstrated by *in vitro* experiments using artificially-created longer-than-wild-type genomes [17], and is possibly also reflected in a general lack of natural over-size insertion mutants reported in the literature. The viral capsid can also be expected to impose an intrinsic restriction on the size of genome that could be packaged within, along with the associated polymerase. Furthermore, studies of hepadnaviral reverse transcription mechanisms have revealed that efficiency of proper polymerase translocation giving rise to correct replication products deteriorates when the distance between 5′ and 3′ DRs exceeds the wild-type genome length [17,18].

Secondly, although initiation of reverse transcription of pgRNA could be mediated *in trans* by polymerase translated from other mRNA, the efficiency has been shown to be far inferior to that initiated *in cis* by polymerase translated from pgRNA [19]. Such tight coupling of polymerase translation with genome replication dictates that recombinant genome needs to encode a functional polymerase for it to be able to replicate with a wild-type level of efficiency and subsequently produce applicable quantities of recombinant virions. Given that coding sequences of wild-type polymerase make up ~75% of the hepadnavirus genome, very limited space is available for engineering purposes if polymerase ORF is to be left untouched, heavily restricting cargo capacity. On the other hand, obliterating polymerase ORF would markedly free up more space for cargo sequences, but at the expense of replication competence.

Thirdly, orthohepadnaviruses encode an X protein (HBx) that has been shown to be capable of playing a myriad of functions in virus-host interactions *in vitro* and/or *in vivo* [20]. Most notably, HBx is apparently a key stimulator of both cccDNA-directed transcription and, indirectly via the regulation of pgRNA synthesis, genome replication [21]. HBx does not appear to be packaged into HBV virions and experiments *in vitro* have demonstrated that HBV mutants lacking functional HBx ORF could not establish or sustain productive infection in susceptible cells [22,23]. Therefore, for a recombinant HBV to be optimally active in infected cells, intact HBx ORF is most likely required. Fortunately, since the entire length of HBx is overlapping with other vital elements of HBV genome (Figure 1), HBx ORF can be easily left untouched in recombinant HBV vector design.

Intrinsic peculiarities of hepadnavirus virology not only impose the above challenges for the design and engineering of recombinant virus vectors, but they also make testing of these vectors technically difficult. For example, the envelopment of hepadnaviral capsids appears to be a highly-specific process that, in distinct contrast to some other enveloped viruses like poxviruses and lentiviruses, does not normally incorporate membrane proteins other than the corresponding hepadnaviral envelope proteins. This makes it highly difficult to create pseudotyped hepadnaviruses that could be tested on cells of non-liver origin. Consequently, testing of recombinant hepadnavirus vectors have to rely on hepatocytes that support infection by wild type hepadnavirus.

For the prototype avihepadnavirus DHBV, effective infection *in vivo* of ducklings and *in vitro* of either primary duck hepatocytes or the chicken hepatoma-derived LMH cell line have been commonly used [3,15]. For HBV, however, stably reproducible infection *in vivo* has only been demonstrated for certain higher primates, especially chimpanzees [15,24], which are economically and ethically prohibitive for routine experiments. Alternatively, chimeric immunodeficient mice harboring human primary hepatocytes can be used to simulate HBV infection *in vivo* [25,26], but in the absence of normal immune functions, virus-host interactions are not fully reflected. *In vitro*, primary human and tree shrew hepatocytes [27], hepatoma-derived HepaRG cells [28] and, recently, liver cell lines stably transfected with the HBV receptor NTCP (Na^+^-taurocholate cotransporting polypeptide) [29], have been used. Although these systems support HBV infection with varying efficiencies, so far only primary infection can be achieved, without demonstrable secondary infection by progeny viruses. For WHV, although woodchucks can be routinely infected using viruses prepared from naturally- or experimentally-infected animals, efficient production of infectious virus *in vitro* has yet to be achieved.

## 4. Hepadnavirus-Derived Viral Vectors

It was more than 25 years ago when the first work dealing with recombinant hepadnaviruses was published [30]. Since then, attempts at engineering usable hepadnavirus vectors have been made repeatedly, with varying degrees of success. Due to its obvious clinical relevance, most efforts have targeted HBV. This section will summarize historical and recent progresses by describing and comparing vector designs validated by sufficient experimental data (Table 1).

As discussed in the previous section, reverse transcription of hepadnaviral pgRNA is far more efficient if it is initiated *in cis* by polymerase translated from the same pgRNA. Most researchers, therefore, have opted to retain polymerase expression in their vector design in the hope of achieving higher replicative competence. Recombinant hepadnavirus designs are, therefore, divided into two categories according to whether functional polymerase is encoded by the vector or needs to be complemented *in trans*, and discussed respectively.

### 4.1. Vectors that Encode Functional Polymerase

Characterization of hepadnavirus polymerase functions revealed early on that it is made up of four structurally and functionally distinct domains: an N-terminal TP (terminal priming) domain that is responsible for catalyzing the priming of negative strand DNA synthesis after binding to pgRNA epsilon *in cis*, a spacer or tether domain that joins the neighboring domains, an RT (reverse transcription) domain that synthesizes both negative and positive strands of the progeny viral genome, and an RH (RNaseH) domain that degrades pgRNA after negative-strand DNA is synthesized [3]. Among the polymerase domains, the spacer is the least conserved and predicted to be the least structurally ordered. Most likely, it only functions as a physical linker or hinge.

#### 4.1.1. Vectors that Use Polymerase Spacer Region for Cargo Insertion

While examining the ability of DHBV polymerase to tolerate mutations and insertions at various locations, Chang *et al.* found that insertion of coding sequences for bacterial protein A (369 nt) without termination codon in-frame into the spacer region, between preS and S in the overlapping ORF, did not abolish polymerase activity and self-sufficient replication of the resultant recombinant genome [30]. The cargo ORF carried its own start codon, making it theoretically possible for preS/S mRNA and probably also pgRNA to translate protein A fused to the C-terminal half of polymerase, in addition to full-length polymerase with protein A sequences embedded in the spacer region, but this former type of fusion protein was apparently not translated to detectable levels in transfected cells. Since the preS/S ORF is interrupted by cargo insertion, the recombinant genome would require *trans-*complemented envelope proteins to form mature progeny virions. Although this early work was not a study devoted to recombinant HBV and recombinant virion production was not tested, the results showed that polymerase spacer is a viable cargo insertion site for engineering self-replicating recombinant hepadnavirus.

The first comprehensive study of recombinant hepadnavirus was published by Chaisomchit *et al.* in 1997 [31] with a design scheme similar to the work by Chang *et al.* The authors proposed recombinant HBV as “more efficient means for gene delivery to the liver” compared to retroviral and adenoviral vectors. They first tested the possibility by inserting coding sequences for HIV Tat protein (267 nt) without a stop codon in-frame into the polymerase spacer, between the Sp1 promoter and preS1 start codon in the overlapping ORF (Figure 1B, design I). This insertion site was located more upstream and closer to the TP domain compared to the protein A insertion site in the previous work. HBV polymerase with Tat sequences inserted in spacer was functional and replicated recombinant genome at efficiencies that were 1.5%–4% of wild-type HBV. As only P ORF is affected, the recombinant genome does not require *trans*-complementation of viral structural proteins and enveloped recombinant virions could be detected in transfection supernatants, but only at very low levels. Infectivity of recombinant virions was not tested. Tat-induced transcription activation of promoters could be detected in transfected cells, and it was shown that Tat fused to C-terminal part of polymerase could be translated from Sp1 transcribed mRNA. This pioneering study demonstrated with compelling evidence that polymerase spacers could tolerate fairly long insertions at the cost of replication efficiency.

Both of these two early studies used similar design that inserted cargo genes into polymerase spacer of wild-type genomes. Our lab also made an attempt to harness the polymerase spacer as an insertion site to develop recombinant HBV vectors for hepatocyte-specific delivery of reporter and functional genes. However, instead of wild-type HBV, we based our design on a clinically-isolated, highly-replicative HBV mutant that harbors a large in-frame deletion of 207 nt in the polymerase spacer [37]. The mutant does not encode functional envelope proteins due to the partial loss of preS1 ORF and non-sense mutations in S ORF, and the polymerase contains a 69 amino acid deletion in the spacer region. However, the mutant replicates more efficiently than the wild-type, and when *trans*-complemented with functional envelope proteins, produces mature enveloped progeny viruses also more efficiently than the wild-type. We inserted terminated ORF encoding the N-terminal activation domain of HIV Tat (207 nt) into the deletion in the polymerase spacer, but unlike the previous two studies, the inserted ORF was in-frame with preS1 so as not to be expressed fused to polymerase, and multiple synonymous mutations were used to avoid terminating the overlapping P ORF (Figure 1B, design II). The recombinant HBV replicated and produced progeny virions with efficiencies comparable to the wild-type, and Tat expression driven by Sp1 could be detected using reporter assay. The polymerase spacer deletion in the mutant was then maximized to increase cargo capacity and we obtained a vector with a 384 nt in-frame deletion that replicated as efficiently as the parental mutant. The vector could tolerate insertions of up to 675 nt and still retain wild-type-level replicative competence, as long as P ORF is not interrupted. We demonstrated that recombinant HBV carrying synonymously-mutated sequences encoding DsRed infected PTH with high efficiency. Moreover, we showed that the vector could carry and express functional RNA in infected PTH, which was the first report of recombinant HBV delivering non-protein cargo. Naturally, the major limitation of this design is that cargo sequences must not introduce stop codons in P ORF, which is often difficult and sometimes impossible.

#### 4.1.2. Vectors that Use Core Region for Cargo Insertion

Inserting cargo sequences into polymerase spacer while keeping the resultant polymerase active is inherently difficult. Consequently, other published designs in this category chose to avoid changing P ORF. On the hepadnaviral genome, all *cis*-acting sequence elements required for genome replication and packaging are clustered closely together between the C-terminal part of polymerase and N-terminal of core, with the remaining part of polymerase taking up almost all of the rest of genome space (Figure 1). The only segment of the genome that is apparently replaceable without affecting P ORF or *cis* elements is the middle part of C ORF (~350 nt) between epsilon packaging/polyadenylation signals and the start codon of P ORF.

In an attempt to test recombinant HBV as a potential liver-targeting delivery vector, Wang *et al.* examined a series of HBV deletion mutants, one of which allowed the replacement of the central part of C ORF with a short terminated ORF encoding Flag tag (48 nt) that was inserted in-frame with the preceding N-terminal of C ORF (Figure 1B, design III) [32]. The recombinant genome could replicate at levels much lower than wild type when *trans*-complemented with core, and produced recombinant virions that infected PHH with low efficiency. The Flag tag is expected to be expressed as a C-terminal fusion to the remaining N-terminal of e and c antigens. However, extending the Flag tag with C-terminal addition of full-length or truncated GFP sequences resulted in a loss of replicative competence. In a similar study roughly coinciding with this work, Yoo *et al.* replaced the same part of C ORF with GFP-encoding sequences (~720 nt), without specifying whether the insertion was in-frame with C or whether it carried its own start and stop codons. Replication efficiency of this recombinant HBV was about 3% of wild-type HBV in the presence of *trans*-complemented core expression [33]. However, no marked increase in genome size was observed in Southern blot as should be expected. Enveloped virions were demonstrated in co-transfection supernatants at significantly reduced levels compared to the wild-type. Recombinant virions infected primary human hepatocytes and questionably, also HepG2, and resulted in detectable fluorescence in infected cells.

These two studies demonstrated that the central part of C ORF is also a viable choice as cargo insertion site, but the reported insertions severely affected replication competence. In wild-type HBV, both core and polymerase are translated from pgRNA. Multiple mechanisms have been shown to be involved in translation initiation of the downstream P ORF, including leaky scanning and ribosome re-initiation [41], and sequences upstream of polymerase start codon significantly affect the translation efficiency of polymerase and consequently, replication efficiency of the genome. In light of this, inefficient expression of polymerase might be responsible, at least partially, for the low replicative competence observed in the two studies discussed above. In a study aimed at liver-targeting delivery of immunogenic peptides, Deng *et al.* attempted to alleviate this problem by optimizing sequences surrounding HBV polymerase start codon according to Kozak’s rules [34], while replacing the upstream central part of C ORF with sequences encoding a polyepitope peptide (180 nt) in a fashion similar to the design of Wang *et al.* above. The recombinant HBV apparently replicated better than wild-type HBV in the presence of a *trans*-complemented core, and in a follow-up study, mature virions infectious for primary tupaia hepatocytes (PTH) were demonstrated to be produced at levels lower than the wild-type [35]. Expression of the cargo peptide, however, was only shown using plasmid or recombinant adenovirus as delivery vectors [34,35].

An alternative approach to enhancing polymerase translation is to make it independent of upstream sequences by inserting an internal ribosome entry site (IRES) before its start codon. In the work by Wang *et al.* [35], the overlapping part of C/P ORFs was duplicated to create non-overlapping C and P ORFs. ORF encoding blastidicin resistance protein (BsdR, 399 nt) or GFP (720 nt) with a termination codon at the 3’ end was then inserted between C and P ORFs, and short IRES units were used to separate the three (Figure 1B, design IV). Since all viral ORFs are still present, such a design requires no *trans*-complementation of wild-type HBV proteins. Recombinant HBV harboring the shorter BsdR replicated and produced progeny virions with efficiencies generally comparable to wild-type HBV, but insertion of the longer GFP resulted in severely reduced replication and nearly undetectable progeny virus secretion. As duplication of part of C ORF increases the genome size beyond that of the wild-type, even without cargo insertion, inability to harbor long insertions is not surprising. Infectivity of the recombinant virus was then demonstrated using HepaRG cells. An analogous strategy has been used in our lab (submitted) to improve the previously-described vector with a maximized deletion in polymerase spacer [37]. A short artificial IRES was placed before the start codon of P ORF containing the maximized deletion in spacer, and cargo sequences replaced the central part of C ORF (Figure 1B, design II′). Unlike the above studies, cargo protein genes carried own start and stop codons and upstream remaining C ORF was terminated to avoid expressing cargo genes as fusions to the remaining N-terminal of core. Recombinant HBV harboring fluorescent and bioluminescent reporters of 375–747 nt replicated, in the presence of *trans**-***complemented core, with varying efficiencies that were mostly comparable to the wild-type, depending on the length and type of the insertion. Enveloped progeny viruses were obtained by providing wild-type core and envelope proteins *in trans* to recombinant genomes and infectivity of recombinant viruses harboring protein or RNA genes was demonstrated using PTH. Compared to its parental vector, the switch to C ORF for insertion allowed freer choice of cargo sequences, while the inheritance of replication-enhancing deletion in polymerase ORF and isolation of polymerase translation through introduction of IRES provided acceptable replication efficiencies for most of the tested cargos.

### 4.2. Vectors that Do Not Encode Functional Polymerase

Theoretically, since all *cis*-acting sequence elements required for genome replication and packaging are located between the C-terminal part of the polymerase and N-terminal of the core (Figure 1), cargo sequences can be inserted anywhere, or replace any segment(s), on the rest of hepadnavirus genome, if polymerase expression does not need to be retained by the recombinant vector. Such vectors would have maximal capacity for harboring cargo sequences. However, all except one of the few reports on vectors belonging to this category chose to use the S or P ORF for cargo insertion, fairly distant from those *cis*-acting elements, which might be beneficial by minimizing interference of their functions by cargo sequences.

In their pioneering work on recombinant HBV vectors, Chaisomchit *et al.* also tested a non-replicative version of their design by replacing the un-terminated in-frame Tat insertion in polymerase spacer with a terminated in-frame insertion of ZeoR (372 nt) (Figure 1B, design I) [31]. Self-sufficient replication of the recombinant genome was expectedly obliterated, and even with *trans*-complemented polymerase, replication was only 1.5%–3% of wild-type HBV, which is similar to the replicative vector with non-terminated Tat insertion. Virion formation and infection were not examined.

Later, in the hope of achieving delivery of therapeutic genes to hepatocytes, Protzer *et al.* developed DHBV and HBV vectors that have most of the S ORF replaced with GFP or duck interferon coding sequences in-frame (Figure 1A, design I and Figure 1B, design V) [17]. The overlapping P ORF is prematurely terminated by the cargo sequences. Recombinant viruses infectious for primary duck or human hepatocytes (PDH and PHH) could be obtained from transfection supernatants, if *trans*-complemented with both polymerase and envelope proteins. Notably, the authors showed that a recombinant DHBV-expressing duck interferon was able to inhibit co-infecting wild-type DHBV in PDH infection assay, which constituted the first demonstration of the therapeutic value of recombinant hepadnavirus. Following up on this work, Untergasser *et al.* attempted to improve the vector from the safety perspective of potential gene therapy applications by prematurely terminating all viral ORFs in the vector so that the recombinant HBV would express only the cargo gene product [38]. Since there was no significant change to the vector design, recombinant HBV harboring GFP or renilla luciferase (942 nt) were obtained when *trans*-complemented wild-type HBV proteins, and the virions infected PHH and expressed the cargo genes. In addition, stronger, exogenous promoters were tested in replacement of Sp2 in the hope of enhancing cargo gene expression, but although recombinant viruses infectious for PHH were produced, enhanced expression was not demonstrated using infection assay. Similar design was used by Liu *et al.* in a later study also aimed at liver-targeting delivery of therapeutic genes [39]. The authors replaced S ORF in-frame with sequences encoding GFP or RFP, and observed reduced replication compared to wild type HBV in the presence of *trans*-complemented HBV proteins. GFP expression by recombinant virions in infected HepaRG was demonstrated.

Recently, Nishitsuji *et al.* [40] reported a system allowing quantitative detection of HBV infection *in vitro* based on a polymerase-negative design of recombinant HBV that uses C ORF for cargo insertion, instead of polymerase or S ORF, as done in the previous designs in this category. However, in contrast to vectors that use C ORF as insertion site in the first category, they replaced the entire C ORF downstream of epsilon packaging/polyadenylation signals (562 nt), including the part overlapping with the N-terminal of P ORF, thus destroying polymerase expression (Figure 1B, design VI). Recombinant HBV harboring the NanoLuc reporter (513 nt) could be produced when *trans*-complementation of core and polymerase were provided and infection of human hepatocytes and NTCP-transfected hepatoma cell lines was demonstrated.

### 4.3. Comparison of Vector Designs from the Perspective of Potential Applications

Potential *in vitro* applications of recombinant hepadnavirus vectors include the study of fundamentals of hepadnavirus life cycle, most notably entry into target cells and formation of initial cccDNA from the incoming virus genome. At the same time, reporter-expressing recombinant hepadnaviruses would facilitate the development of drug screening and evaluating systems. *In vivo*, recombinant hepadnaviruses could form the basis of hepatocyte-targeting therapeutic interventions for liver-afflicting conditions, including HBV-related and non-HBV-related hepatitis, cirrhosis, and HCC, as well as life-threatening diseases not directly involving liver, such as type I diabetes. Reporter-expressing recombinant HBV would be useful for characterizing hepatotropism, sustenance, and biosafety of such therapeutics before *in vivo* applications could be attempted.

Different applications of recombinant virus vectors sometimes have different requirements regarding the vectors, in addition to functions of the cargo gene. For instance, for therapeutic interventions targeting chronic hepatitis B patients using recombinant HBV expressing interferon or HBV-targeting siRNA precursors, continuous high activity of a recombinant virus is only desirable while wild-type HBV is active in co-infected hepatocytes, but not thereafter. In contrast, sustained activity of recombinant HBV capable of surviving hepatocyte propagation and turnover, regardless of the presence or absence of wild-type HBV, is desired when, for example, using insulin-expressing recombinant HBV for treating type I diabetes.

Features of the recombinant hepadnavirus vector designs reviewed above are summarized in Table 2 with a focus on their capabilities of self-sufficient replication and progeny virus production. Recombinant hepadnaviruses encoding both functional core and polymerase proteins [31,36,37] are expected to be able to replicate their genomes and expand intranuclear cccDNA pools resulting in sustained high expression of cargo genes in infected cells, irrespective of wild-type co-infection. Consequently, such vectors would persist in infected hepatocytes and are, therefore, ideal for *in vivo* applications targeting non-HBV-related life-long diseases, and for studies where highly-sensitive detection of infection is desired. Progeny recombinant viruses will also be produced if core- and polymerase-producing recombinant viruses also encode functional envelope proteins [31,36]. This would theoretically allow secondary infection by progeny recombinant viruses of surrounding susceptible cells, further enhancing the level and continuity of cargo gene expression.

Loss of functional core and/or polymerase expression makes the majority of recombinant vectors incapable of replication, and consequently, progeny production, without co-infecting wild-type virus (Table 2). These vectors would likely persist with low activity and no expansion in mono-infected cells, but would start replication and progeny virus production, giving rise to enhanced cargo gene expression and expanded infection by recombinant virus, if the infected cells are to be super-infected with wild-type virus. Activity of recombinant virus will recede along with wild-type virus, if and when the latter is under control. Such activation by the wild-type makes these vectors ideal for therapeutic applications targeting infection by wild-type viruses.

## 5. Future Perspectives on Recombinant Hepadnavirus Vector Design

Minimal requirements for hepadnavirus genome replications include *cis* elements on pgRNA (Figure 1), functional core *in cis* or *in trans*, and functional polymerase preferably expressed *in cis*. Designs that do not retain functional polymerase on the recombinant genome theoretically would allow much freer choice of cargo length and insertion site. However, published studies in this category have not demonstrated such potential, and are generally limited to using fairly short cargo genes to replace viral ORF (Table 1). It is possible that future work on such vectors will liberate additional viral genome space and sites for cargo insertions, and enable the design of recombinant viruses harboring larger genes or multiple small genes to provide better or more complex functionalities.

Recombinant hepadnavirus designs that retain functional polymerase have inherently limited capacity for cargo insertions. Even with non-inactivating deletions in polymerase spacer [37], 600–700 nt is very likely the maximum cargo length that is practical for HBV. The capacity will be further reduced if functional core is also to be retained [31,36]. However, the relative ease of recombinant virus production and expected higher expression of cargo genes in infected cells compared to polymerase-negative designs make such designs a favored choice in most cases. Finding or engineering small-sized cargo genes with experimentally- or clinically-important functions will be the key to making these vector designs more relevant to the field.

## 6. Future Perspectives on Applications of Recombinant Hepadnavirus Vector

Work on developing recombinant hepadnavirus vectors has been going on for two decades and there have been more than ten designs with varying degrees of similarity and innovation (Table 1). Some of the studies characterized the cargo capacity, replication, progeny virus production and infectivity in fairly great detail and convincingly demonstrated the usability of the corresponding vector, at least when used with the tested cargo. A couple of studies went further and showed the huge potential of such vectors for possible therapeutic applications *in vivo* [17,37].

Admittedly, compared to other more commonly used recombinant viral vector systems, such as retrovirus/lentivirus, poxvirus, adenovirus, and adeno-associated virus vectors, progress in the development and application of recombinant hepadnaviruses both *in vivo* and *in vitro* has been much slower and less fruitful. Nevertheless, lack of strict hepatotropism makes the other recombinant virus vectors intrinsically inferior to hepadnavirus vectors for hepatocyte-targeting applications. Moreover, for potential applications in chronic HBV-infected patients, recombinant HBV is minimally affected by vector-targeting immune reactions, in distinct contrast to other virus vectors. Needless to say, for studying basic virology of hepadnaviruses, only recombinant hepadnaviruses are irreplaceable tools for obtaining meaningful results. So far, however, studies that actually take advantage of the reported recombinant hepadnavirus vector systems to address unanswered virological questions and unmet clinical demands have been rare and mostly restricted to labs that originally developed the systems. The reasons behind such apparent lack of application are manifold.

Firstly, large-scale preparation of recombinant viruses is a very cumbersome process, especially for poorly replicative constructs. The use of stronger promoter, such as CMV promoter, instead of native Cp promoter to drive pgRNA transcription could enhance, to a limited degree, the production of progeny viruses. In future, identifying and counteracting cellular mechanisms restricting hepadnaviral replication might give rise to novel production systems. The necessity of providing *trans-*complementation of viral proteins for most designs further complicates production and may also incur the risk of wild-type contamination through homologous recombination [38], the mitigation of which would require extensive synonymous mutations. Engineering stably-transfected cell lines that continuously produce recombinant viruses at acceptable levels in the supernatants will significantly boost adoption and application of recombinant hepadnavirus vectors.

Secondly, infection systems for HBV used to be tedious and costly to establish and use, especially for *in vivo* applications. The identification of NTCP receptor for HBV and demonstration that NTCP-overexpressing hepatoma cell lines supporting wild-type and recombinant HBV infection no doubt represent a significant advance in this respect [29]. With the advent and general availability of easier-to-handle *in vitro* HBV infection systems, like HepG2/NTCP [29], interest in and applications of viable recombinant hepadnavirus vectors can be expected to grow. It is also possible that with further understanding of HBV infection mechanisms, which could be significantly promoted by the use of reporter-expressing recombinant HBV, transgenic mice supporting HBV infection might be eventually obtained. In addition, advances in WHV reverse genetics might also enable the development of recombinant WHV vectors usable in this important model of HBV.

Thirdly, most reported recombinant hepadnavirus vectors have been demonstrated using only one or two cargo genes (Table 1) and only our lab’s work has used non-protein cargos [37]. Such limited demonstration of recombinant hepadnaviruses’ capability does not help in attracting potentially interested researchers. There are, of course, inherent restrictions on possible choices of cargo genes (see previous sections), but extensive and in-depth characterization of existing vectors for their ability to deliver commonly used, as well as novel cargo sequences with relevant functions, will surely encourage and facilitate wider applications of recombinant hepadnaviruses in both laboratory and clinical settings.

Last, but not least, integration of hepadnavirus sequences into hepatocyte genomes is often detected in chronically-infected subjects and has been linked, at least in some studies, to HCC development [1,2,3]. Unlike retroviruses and lentiviruses, integration of viral genome into a host chromosome is not a necessary step in hepadnavirus life cycle, and probably represents an opportunistic event during the long-term presence and activity of hepadnaviruses in hepatocytes. Unfortunately, fairly limited information is available on the integration mechanisms, as well as preferred integration sites or lack thereof. Similarly, a potential link to HCC has also been proposed for HBx protein without detailed understanding of the underlying molecular details [1,20]. These constitute a major safety concern for any potential *in vivo* applications of recombinant HBV for subjects not chronically infected with HBV. Further understanding of hepadnavirus integration and HBx functions, which could be aided by studies using recombinant hepadnaviruses *in vivo* and *in vitro*, might eventually enable more realistic evaluation of the associated risks and allow recombinant HBV-mediated gene delivery to be applicable to more patients.

## Figures and Tables

**Table 1:** Listing and comparison of recombinant hepadnavirus vector design.

Publication	Virus Base	Required *trans-*Complementation ^1^	Cargo Insertion Site and Insertion Strategy	Tested Cargo(s) (Length)	Replication Evidence	Replication Efficiency	Virion Formation Evidence ^2^	Virion Formation Efficiency	Virion Infectivity Evidence
*Recombinant vectors that express functional polymerase*									
Chang *et al.*, 1990 [30]	DHBV	preS/S	Cargo ORF inserted in-frame in Pol spacer between preS and S with own ATG but no stop codon	Protein A (369)	EPA, Southern blot	Comparable	N.D.	N.D.	N.D.
Chaisomchit *et al.*, 1997 [31]	HBV	None	Cargo ORF inserted in-frame in Pol spacer between Sp1 and preS1 start codon with own start but no stop codon	HIV Tat (267)	EPA	Severely reduced	Capture by S antibodies followed by Southern blot	Severely reduced	N.D.
Wang *et al.*, 2002 [32]	HBV	C	Cargo ORF replaced C ORF between ε/A(n) and Pol start codon, fused to remaining N-terminal of C	Flag (48)	Southern blot	Severely reduced	Density gradient ultracentrifuge followed by Southern blot and PCR	N.D.	PHH
Yoo *et al.*, 2002 [33]	HBV	C	Cargo ORF replaced C ORF between ε/A(n) and Pol start codon	GFP	EPA, Southern blot	Severely reduced	Density gradient ultracentrifuge and capture by S antibodies followed by Southern blot	N.D.	PHH, HepG2 ^3^
Deng *et al.*, 2009 [34] Wang *et al.*, 2014 [35]	HBV	C	Cargo ORF replaced C ORF between ε/A(n) and Pol start codon, fused to remaining N-terminal of C. Kozak sequences of Pol were optimized.	Peptide (180)	Southern blot	Increased	Density gradient ultracentrifuge followed by dot blot	Reduced	PTH
Wang *et al.*, 2013 [36]	HBV	None	Cargo ORF inserted between separated C and Pol ORF with intervening short IRES	BsdR (399) GFP (720)	EPA, Southern blot	Comparable to severely reduced depending on cargo	Density gradient ultracentrifuge followed by Southern blot	Comparable to severely reduced depending on cargo	HepaRG
Hong *et al.*, 2013 [37]	HBV	preS1/preS2/S	Cargo sequences replaced 384 bp of Pol spacer (preS1/preS2) in-frame with preS1, without terminating Pol ORF. Start codon of preS1 mutated.	HIV Tat (207) DsRed (678) shRNA cassette (294)	Southern blot	Comparable to reduced depending on cargo	Capture by preS1 mAb followed by Southern blot	Comparable to reduced depending on cargo	PTH
Bai *et al.*, (submitted)	HBV	C preS1/preS2/S	Cargo sequences replaced C ORF between ε/A(n) and Pol start codon. Short IRES placed before Pol start codon and 384 bp of Pol spacer (preS1/preS2), same as above, deleted. C ORF prematurely terminated.	ZeoR (375) NanoLuc (522/606) ^4^ DsRed (678) GFP (747) shRNA cassette (294)	Southern blot	Comparable to reduced depending on cargo	Capture by preS1 mAb followed by Southern blot	Comparable to reduced depending on cargo	PTH
*Recombinant vectors that do not express functional polymerase*																		
Chaisomchit *et al.*, 1997 [31]	HBV	Pol	Cargo ORF inserted in-frame in Pol spacer between Sp1 and preS1 start codon with own start and stop codon	ZeoR (372)	EPA	Severely reduced	N.D.	N.D.	N.D.
Protzer *et al.*, 1999 [17]	DHBV	Pol preS/S	Cargo ORF replaced 558 bp of S ORF in-frame	GFP (733) Duck IFN (591)	N.D.	N.D.	Density gradient ultracentrifuge followed by dot blot	N.D.	PDH
*Ibid.*	HBV	Pol preS1/preS2/S	Cargo ORF replaced 939 bp of S ORF in-frame	GFP (733)	N.D.	N.D.	*Ibid.*	N.D.	PHH
Untergasser *et al.*, 2004 [38]	HBV	All HBV ORFs	Cargo ORF replaced 939 nt of S ORF in-frame. All other HBV ORFs are prematurely terminated by mutation. Some constructs replaced ~311 nt of SP2 with exogenous promoters 366 nt or 575 nt long.	GFP (733) RLuc (942)	Southern blot	N.D.	*Ibid.*	Comparable	PHH
Liu *et al.*, 2013 [39]	HBV	All HBV ORFs	Cargo ORF replaced S ORF in-frame. All other HBV ORFs are prematurely terminated or nulled by mutation.	GFP RFP	Southern blot	Reduced	N.D.	N.D.	HepaRG
Nishitsuji *et al.*, 2015 [40]	HBV	Pol C	Cargo ORF with own start and stop codons replaced 562 nt of C ORF downstream of ε/A(n) and the N-terminal of P ORF	NanoLuc (513)	N.D.	N.D.	Density gradient ultracentrifuge followed by Southern blot	N.D.	PXB NTCP cell lines

^1^ core and polymerase are absolutely required for genome replication; envelope proteins (DHBV preS/S and HBV preS1/preS2/S) are only required for virion formation. ^2^ due to secretion of non-enveloped capsids by transfected cells [35,36,37,40], detection of viral DNA in transfection supernatants without virion-specific separation or enrichment step(s) is considered only evidence of replication. ^3^ despite early controversies, human hepatoma cells lines, such as HepG2 and Huh-7, are currently generally accepted as not susceptible to HBV infection. ^4^ two forms, one intracellular and one secreted, were tested. ε, signal on pre-genomic RNA. A(n), polyadenylation signal. EPA, endogenous polymerase activity assay, which measures polymerase-catalyzed incorporation of isotope-labelled nucleotides into progeny genomes within viral capsids. PHH, PTH, and PDH refer to primary human, tupaia, and duck hepatocytes, respectively. PXB, hepatocytes prepared from chimeric mouse harboring human primary hepatocytes. N.D., not done or not shown.

**Table 2:** Comparison of recombinant hepadnavirus vector designs.

Representative Publication(s)	Obliterated ORF(s)	cccDNA Pool Expansion ^1^	Progeny Virus Production ^2^
Chaisomchit *et al.,* 1997 [31]	None	Self-sufficient	Self-sufficient
Wang *et al.,* 2013 [36]			
Hong *et al.,* 2013 [37]	S	Self-sufficient	Requires help
Wang *et al.,* 2002 [32]	C	Requires help	Requires help
Yoo *et al.,* 2002 [33]			
Deng *et al.,* 2009 [34]			
Wang *et al.,* 2014 [35]			
Bai *et al.* (submitted)	C/S	Requires help	Requires help
Chaisomchit *et al.,* 1997 [31]	P	Requires help	Requires help
Protzer *et al.,* 1999 [17]	P/S	Requires help	Requires help
Chang *et al.,* 1990 [30]			
Nishitsuji *et al.,* 2015 [40]	P/C	Requires help	Requires help
Untergasser *et al.,* 2004 [38]	All	Requires help	Requires help
Liu *et al.,* 2013 [39]			

^1^ vectors with functional C and P ORFs are expected to be able to replicate self-sufficiently and form additional cccDNA in infected cells which would, in turn, result in higher cargo gene expression. ^2^ vectors retaining all functional viral ORFs are expected to be able to replicate self-sufficiently and produce infectious progeny recombinant viruses, which would in turn result in infection of additional susceptible cells. Requires help: *trans*-complementation of obliterated proteins by a co-infecting wild-type virus is required for indicated functions.
